# A prospective, observational study of frailty, quality of life and dialysis in older people with advanced chronic kidney disease

**DOI:** 10.1186/s12877-023-04365-4

**Published:** 2023-10-16

**Authors:** Shannon J. King, Natasha Reid, Sarah J. Brown, Lucinda J. Brodie, Aaron D. H. Sia, Mark D. Chatfield, Ross S. Francis, Nancye M. Peel, Emily H. Gordon, Ruth E. Hubbard

**Affiliations:** 1 Centre for Health Services Research, Faculty of Medicine, The University of Queensland, St Lucia, QLD Australia; 2 Western Australian Country Health Service, Busselton Health Campus, West Busselton, WA 6280 Australia; 3 Cairns and Hinterland Hospital and Health Service, Brisbane City, QLD Australia; 4 Department of Kidney and Transplantations Services, Princess Alexandra Hospital, Woolloongabba, QLD Australia; 5 Department of Geriatric Medicine, Princess Alexandra Hospital, Woolloongabba, QLD Australia

**Keywords:** Renal insufficiency, chronic, Renal dialysis, Frailty, Mortality, Quality of life

## Abstract

**Background:**

Frailty is prevalent in older people with chronic kidney disease (CKD) and robust evidence supporting the benefit of dialysis in this setting is lacking. We aimed to measure frailty and quality of life (QOL) longitudinally in older people with advanced CKD and assess the impact of dialysis initiation on frailty, QOL and mortality.

**Methods:**

Outpatients aged ≥65 with an eGFR ≤ 20ml/minute/1.73m^2^ were enrolled in a prospective observational study and followed up four years later. Frailty status was measured using a Frailty Index (FI), and QOL was evaluated using the EuroQol 5D-5L instrument. Mortality and dialysis status were determined through inspection of electronic records.

**Results:**

Ninety-eight participants were enrolled. Between enrolment and follow-up, 36% of participants commenced dialysis and 59% died. Frailty prevalence increased from 47% at baseline to 86% at follow-up (change in median FI = 0.22, *p* < 0.001). Initiating dialysis was not significantly associated with change in FI. QOL declined from baseline to follow-up (mean EQ-5D-5L visual analogue score of 70 vs 63, *p* = 0.034), though commencing dialysis was associated with less decline in QOL. Each 0.1 increment in baseline FI was associated with 59% increased mortality hazard (HR = 1.59, 95%CI = 1.20 to 2.12, *p* = 0.001), and commencing dialysis was associated with 59% reduction in mortality hazard (HR = 0.41, 95%CI = 0.20 to 0.87, *p* = 0.020) irrespective of baseline FI.

**Conclusions:**

Frailty increased substantially over four years, and higher baseline frailty was associated with greater mortality. Commencing dialysis did not affect the trajectory of FI but positively influenced the trajectory of QOL from baseline to follow-up. Within the limitations of small sample size, our data suggests that frail participants received similar survival benefit from dialysis as non-frail participants.

**Supplementary Information:**

The online version contains supplementary material available at 10.1186/s12877-023-04365-4.

## Introduction

Frailty is highly prevalent in chronic kidney disease (CKD), with reported rates up to 43% in patients with advanced CKD [1], and 82% in patients receiving maintenance haemodialysis [2]. Frailty captures variation in health status of people of the same chronological age. It is conceptualised as a state of increased vulnerability to stressors, representing a multidimensional syndrome manifesting clinically with deficits across many domains including cognition, function, sensorium and mood [3, 4]. The coexistence of frailty and CKD is considered to be related to shared pathophysiological mechanisms including inflammation, protein catabolism, mitochondrial dysfunction and oxidative stress [5]. Frailty in patients with CKD predicts risk of adverse outcomes including increased mortality [2, 6], hospitalisation [7, 8], falls [8], and decreased quality of life [9, 10].

In community-dwelling populations, it is understood that frailty is a dynamic state: frailty may decrease with targeted interventions or with resolution of acute stressors, or it may increase with the onset of acute stressors or the passing of time [11, 12]. There is limited prospective data regarding frailty transitions in CKD. In particular, it is unclear how initiating dialysis impacts frailty trajectories, mortality and other outcomes in frail older adults. In Australia and New Zealand, 20% of patients initiating dialysis are aged 75 years and older [13], yet routine identification of frailty status prior to dialysis initiation is lacking [14]. There is a paucity of high quality evidence regarding the benefits, risks and burden of dialysis compared to conservative management in *frail* older patients with kidney failure. This limited evidence base makes balancing patient, carer, clinician, and health service priorities challenging [15–17].

Despite the known associations between frailty and adverse outcomes in patients with CKD, no published prospective studies to date have examined the interaction between commencement of dialysis and frailty, quality of life and mortality. Our primary study aims were to measure frailty and quality of life prospectively in older patients with advanced CKD and to assess the impact of dialysis initiation on frailty, quality of life and mortality.

## Methods

Strengthening the Reporting of Observational Studies in Epidemiology (STROBE) guidelines were adhered to when preparing this manuscript [18].

### Study design, setting and participants

This prospective single-centre observational study enrolled participants presenting to an outpatient advanced kidney disease clinic in a single large tertiary hospital located in Brisbane, Queensland. Every outpatient aged ≥65 yearswho was being followed up by this clinic as at July 2017, had not yet initiated kidney replacement therapy and had an estimated glomerular filtration rate (eGFR) ≤ 20ml/minute/1.73 m^2^ was invited to participate in the study. The only exclusion criterion was inability to communicate in English with no interpreter available.

Written informed consent was obtained from participants prior to enrolment. If required, consent was obtained from their legally authorised representative. Baseline data were collected from July 2017 to August 2018 and follow-up assessments were conducted in July 2022.

Ethics approval for this study was obtained from the Ethics Research Committee in the Metro South Health District (HREC/QPAH-16/649).

### Variables, data sources and measurement

Enrolled participants were interviewed in-person at baseline and via phone call at follow-up. Interviews were conducted by study personnel (SK and SB) who were undertaking specialist training in geriatric medicine. Caregivers were interviewed as a proxy when participants were unable to complete meaningful evaluations due to significant cognitive impairment, which was determined at the time of the assessment by SK or SB.

#### Baseline characteristics

A comprehensive assessment was performed through interview with participants or caregivers at baseline and follow-up using the interRAI Community Health Assessment (CHA) instrument. Baseline variables obtained via self-report included age, sex, country of birth, living arrangements, recent hospitalisation and any comorbid medical conditions. Studies have demonstrated no significant difference between in-person and over-the phone InterRAI assessments [19, 20].

#### Frailty index

Frailty was measured at baseline and follow-up via a frailty index (FI) derived from the interRAI CHA utilising questions and clinical observations related to health, functional and psychosocial characteristics including cognition, communication, mood, activities of daily living, continence and falls. Derivation of FI from interRAI core data items has previously been demonstrated as a valid measure of health status [21], and using an FI to measure frailty has been shown to have good construct validity in patients with CKD [22]. Thirty-two variables were coded as either binary (i.e. 1 = deficit present, 0 = absent) or graded (e.g., 1 = dependent, 0.5 = assistance required, 0 = independent) deficits. Additionally, the number of disease diagnoses (1-point for each diagnosis up to 10) and number of regular medications (scored from 0–3 depending on number of medications) were included, resulting in a denominator of 45. To calculate the FI, the total number of deficits were divided by the denominator. Higher FI indicates greater frailty, with a theoretical maximum of 1. An FI of ≥ 0.25 has been determined to optimally represent the presence of frailty in community-dwelling older adults [23, 24].

#### Quality of life

Health-related quality of life (QOL) was measured at baseline and follow-up via the EuroQoL 5-Dimension 5-Level (EQ-5D-5L) instrument. The EQ-5D-5L is a widely utilised, valid and reliable measure of health status [25]. Health status is assessed via self-report across five domains: mobility, self-care, usual activities, pain/discomfort and anxiety/depression [25]. It also includes a self-reported health visual analogue scale (EQ-VAS) ranked from 0 (the worst health imaginable) to 100 (the best imaginable health) [25]. The EQ-5D-5L has been validated in older people and is widely used in the CKD population [26, 27]. Comparable results between EQ-5D values obtained in-person and over-the phone have been demonstrated [28].

Index values were calculated from the five domain scores using the English dataset as no Australian dataset currently exists [29]. Higher values indicate higher QOL (range -0.285 to 1) [25]. However, due to commonalities between the five EQ-5D-5L domains and deficit variables included in the FI, most of our statistical analyses involving QOL utilised the EQ-VAS.

#### eGFR, dialysis status and mortality status

Information regarding baseline eGFR, date of commencement of dialysis and mortality status/date of death were obtained from an integrated electronic medical record.

### Study size

This was a convenience sample, and no a-priori determination of optimal study size was calculated.

### Statistical analyses

Participants who were lost to follow-up were included in all analyses aside from comparison of baseline to follow-up FI and QOL, as data on dialysis initiation and mortality status were available. Where a proxy completed the EQ-5D-5L rather than the participant, these QOL results were excluded from analysis, due to the lack of association between participant and proxy ratings [30]. Participants who received a transplant were excluded from follow-up analyses.

Continuous variables were compared using two-sample t-tests or Wilcoxon rank-sum test for normally and non-normally distributed variables, respectively. Categorical variables were compared using Pearson’s chi-squared test or Fisher’s exact test.

Wilcoxon signed-rank tests were used to assess absolute change in FI and the EQ-VAS from baseline to follow-up. Natural log transformation of FI and EQ-VAS was used to calculate a percentage change score: 100(ln follow-up – ln baseline)% [31]. Linear regression was used to examine predictors of percent change.

Follow-up person-years were calculated from baseline assessment date to either date of death, date of loss to follow-up or date of follow-up assessment, whichever came first. Kaplan–Meier curves and a log-rank test were used to compare survival by baseline frailty status [32]. A multivariable Cox proportional hazards model (adjusted for age and sex) were used to estimate the association of ordinal FI and/or EQ-VAS and all-cause mortality, accounting for covariates of interest [33]. Dialysis status was treated as a time-varying predictor based on when the participant commenced dialysis, therefore data were split into time before commencement of dialysis and time after commencement of dialysis for assessment of this predictor.

Analyses were performed using Stata (version 17, StataCorp, California, USA) and assumed a 5% significance level.

## Results

### Overview of study recruitment

**Fig. 1:**
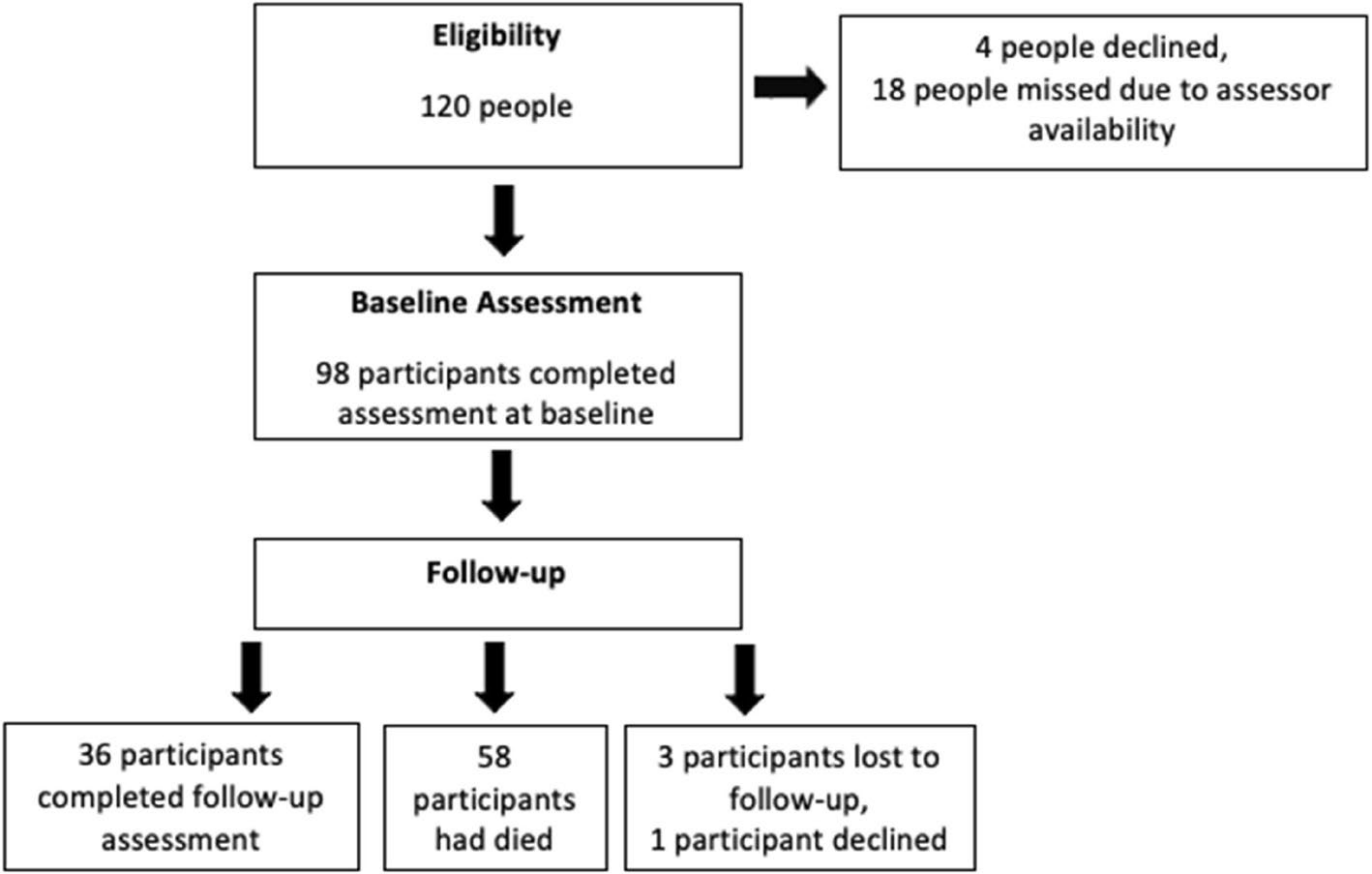
Study flowchart

At the beginning of the study period, 120 people were identified as eligible for inclusion. Four people did not consent to the study, and 18 people were missed due to assessor availability resulting in a total sample of 98 participants at baseline. After a mean follow-up period of 4.2 years; 58 participants (59%) had died, three participants were unable to be contacted and one participant declined; resulting in 36 participants completing follow-up assessment (Fig. 1). Three participants were unable to complete the EQ-5D-5L due to cognitive impairment, otherwise there was no missing data from any participant who consented to baseline and follow-up assessment.

### Participant characteristics

Table 1 summarises baseline participant characteristics. The mean (SD) age of participants at baseline was 76.3 (7.3) years old. A total of 44% were women, 52% were born in Australia, 6% lived in a residential aged care facility and 29% had been hospitalised within the previous 90 days. The median (IQR) eGFR at baseline was 15 (11–17) mL/min/1.73m^2^ with a range of 3–20 mL/min/1.73m^2^.

**Table 1:** Baseline participant characteristics

	Total
	*n* = 98
Characteristics	
Age, mean (SD) (years)	76.3 (7.3)
Sex, n (%)	
Male	54 (55%)
Female	44 (45%)
Country of birth, n (%)	
Other	47 (48%)
Australia	51 (52%)
Living arrangement, n (%)	
Alone	30 (31%)
With others	62 (63%)
RACF	6 (6%)
Hospitalised previous 90 days, n (%)	
Yes	28 (29%)
No	70 (71%)
eGFR, median (IQR)	15 (11–17)
eGFR < 15, n (%)	47 (48%)
Number of comorbidities, mean (SD)	5.2 (1.9)
Number of medications, mean (SD)	9.8 (4.5)
BMI, mean (SD)	28.2 (6.1)
Recent weight loss, n (%)	10 (10%)
Frailty index, median (IQR)	0.24 (0.18–0.31)
Frail (FI ≥ 0.25), n (%)	46 (47%)
EQ-VAS, mean (SD)	65.4 (18.9)
EQ index value, median (IQR)	1.0 (0.80–1.00)

*Abbreviations*: *RACF* residential aged-care facility, *eGFR* estimated glomerular filtration rate in mL/min/1.73m^2^, *BMI* body mass index, *EQ-VAS* EuroQol 5D-5L visual analogue scale, *EQ* index value, EuroQol 5D-5L index value

During the follow-up period, 35 participants (36%) commenced dialysis (24 participants commenced haemodialysis and 11 commenced peritoneal dialysis) and one participant received a kidney transplant. Table 2 outlines baseline characteristics according to dialysis status at end of follow-up. Compared to participants who did not commence dialysis, participants who commenced dialysis were younger (mean age 72.9 vs. 78.4 years, *p* < 0.001), had a lower eGFR (mean 13 vs. 16 mL/min/1.73m^2^, *p* = 0.010) and were more likely to live with others (83% vs 52%, *p* = 0.006). When FI at baseline was measured as a continuous variable, there was no statistically significant difference between dialysis and no dialysis groups (median FI 0.26 vs. 0.21, *p* = 0.068). When analysed as a dichotomous variable (frail/non-frail), there was a significant difference, with a lower percentage of frail participants in the dialysis group compared to the no dialysis group (31% vs. 55%, *p* = 0.026). Quality of life at baseline, measured by the EQ-5D-5L index value, was lower in participants who did not commence dialysis versus those who did (median 0.93 vs. 1.0, *p* = 0.010), though there was no significant difference in quality of life as measured by the EQ-VAS (mean 66.8 vs. 62.5, *p* = 0.29).

**Table 2:** Participant characteristics at baseline according to subsequent dialysis status

	No Dialysis	Dialysis	*p*-value
	*n* = 62	*n* = 35	
Characteristics			
Age, mean (SD) (years)	78.4 (7.4)	72.9 (5.5)	< 0.001
Sex, n (%)			0.83
Male	34 (55%)	20 (57%)	
Female	28 (45%)	15 (43%)	
Country of birth, n (%)			0.69
Other	31 (50%)	16 (46%)	
Australia	31 (50%)	19 (54%)	
Living arrangement, n (%)			
Alone	24 (39%)	6 (17%)	
With others	32 (52%)	29 (83%)	0.006
RACF	6 (10%)	0 (0%)	
Hospitalised previous 90 days, n (%)			0.68
Yes	17 (27%)	11 (31%)	
No	45 (73%)	24 (67%)	
eGFR, median (IQR)	16 (11–18)	13 (10–16)	0.010
eGFR < 15, n (%)	24 (39%)	23 (66%)	0.011
Number of comorbidities, mean (SD)	5.2 (1.9)	5.2 (2.0)	0.95
Number of medications, mean (SD)	10.0 (4.6)	9.5 (4.2)	0.61
BMI, mean (SD)	28.3 (5.9)	27.5 (6.0)	0.53
Recent weight loss, n (%)	7 (11%)	3 (9%)	0.67
Frailty index, median (IQR)	0.26 (0.19–0.32)	0.21 (0.13–0.29)	0.068
Frail (FI ≥ 0.25), n (%)	34 (55%)	11 (31%)	0.026
EQ-VAS, mean (SD)	66.8 (17.7)	62.5 (20.9)	0.29
EQ index value, median (IQR)	0.93 (0.79–1.00)	1.00 (0.94–1.00)	0.010

*Abbreviations*: *RACF* residential aged-care facility, *eGFR* estimated glomerular filtration rate in mL/min/1.73m^2^, *BMI* body mass index, *EQ-VAS* EuroQol 5D-5L visual analogue scale, *EQ* index value, EuroQol 5D-5L index value

### Frailty

**Fig. 2:**
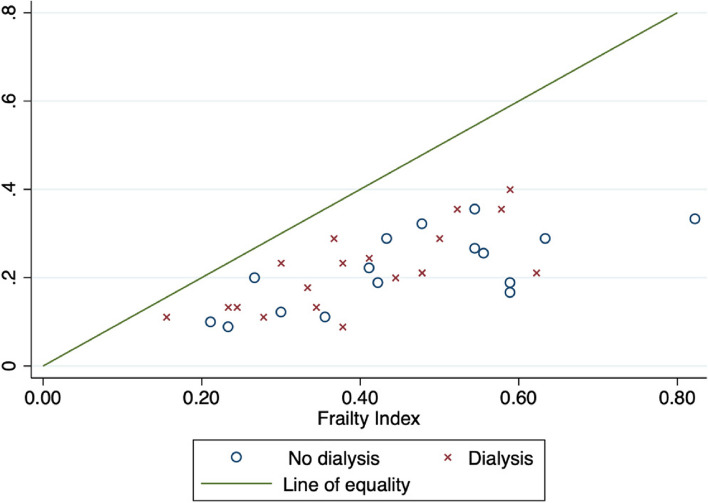
Frailty index at baseline and follow-up

Baseline prevalence of frailty (FI ≥ 0.25) of the whole sample was 47%, with a median (IQR) frailty index of 0.24 (0.18–0.31) and range 0.09 to 0.58. For participants evaluated at follow-up (*n* = 35), their baseline median (IQR) FI was 0.21 (0.13–0.29) and this increased significantly (*p* < 0.001) at follow-up to a median (IQR) FI of 0.43 (0.32–0.54), range 0.16–0.82 and frailty prevalence of 86%. The median change in FI of participants at follow-up of 0.22 represents accumulation of almost 10 additional deficits over the follow-up period. Every participant assessed at follow-up had a higher FI compared to baseline. The scatterplot in Fig. 2 displays the frailty index at baseline and follow-up for the evaluated sample at follow-up (*n* = 35).

Age, sex, dialysis status, number of days on dialysis, and baseline eGFR and EQ-VAS did not predict percent change in frailty index from baseline to follow-up (Supplementary Table 1).

### Quality of life

Quality of life, assessed by EQ-VAS (scale 0–100), declined for participants assessed at follow-up (mean 70.0 vs. 63.0, *p* = 0.034, *n* = 32). Age, sex, dialysis status, number of days on dialysis, and baseline eGFR and frailty index were evaluated as predictors of percent change in EQ-VAS (Supplementary Table 1). Overall, the mean percent change in EQ-VAS amongst participants assessed at follow-up was -10.4%. Participants who commenced dialysis had a mean + 2.5% change in EQ-VAS, while those that did not commence dialysis showed a mean -27% change in EQ-VAS with a between-group difference in mean percentage change [95%CI] = 29% [0.1% to 59%], *p* = 0.049. Each 1mL/min/1.73m^2^ higher baseline eGFR was associated with a mean -6% change in EQ-VAS from baseline to follow-up [95%CI = -11% to -1.5%, *p* = 0.009].

### Mortality

**Fig. 3:**
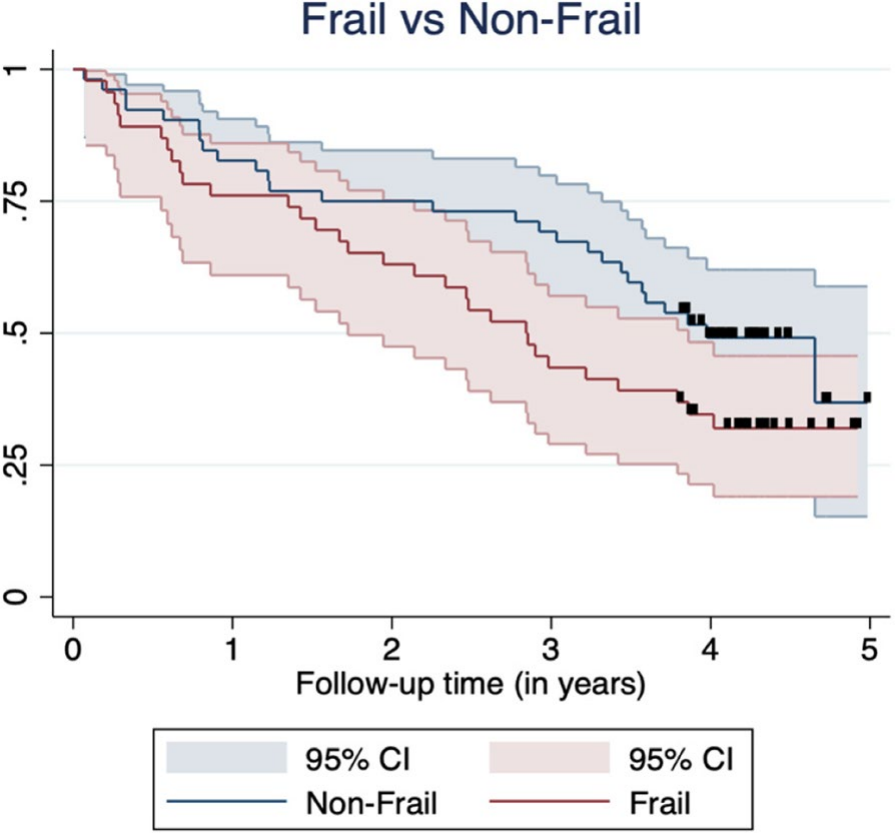
Kaplan–Meier survival curve by frailty category

Fifty-eight participants (59%) died during the follow-up period. Overall median survival time in this cohort was found to be 3.48 years [95%CI = 2.85 to 4.65 years]. A Kaplan–Meier survival curve of frailty (frail vs. non-frail) categories is presented in Fig. 3. Non-frail individuals had longer median survival (3.98 years [95%CI = 3.32, Q3 not reached]) than frail participants (2.84 years [95%CI = 1.72 to 3.86 years]) though this did not reach statistical significance (*p* = 0.054).

A Cox model (Table 3) revealed that each 0.1 increment in FI was associated with a 59% higher hazard for mortality (HR = 1.59, 95%CI = 1.20 to 2.12, *p* = 0.001). Each 1mL/min/1.73m^2^ higher baseline eGFR was associated with a 10% lower hazard for mortality (HR = 0.90, 95%CI = 0.84 to 0.97, *p* = 0.003). Dialysis was associated with a 59% reduction in mortality risk during the study period (HR = 0.41, 95%CI = 0.20 to 0.87, *p* = 0.020). A frailty index (continuous in 0.1 intervals) by dialysis status interaction was not significant (*p* = 0.731) suggesting that the relationship between dialysis and reduced risk of mortality did not differ depending on baseline frailty index.

**Table 3:** Multivariable cox regression for mortality

	**HR**	**95% CI**	***p*-value**
Baseline FI (increasing 0.1 intervals)	1.59	1.20, 2.12	0.001
Baseline EQ-VAS	0.99	0.98, 1.01	0.363
Baseline eGFR	0.90	0.84, 0.97	0.003
Dialysis^a^	0.41	0.20, 0.87	0.020
Dialysis^a^ x FI (increasing 0.1 intervals)	0.87	0.40, 1.89	0.731

All analyses included age and sex as covariates. Baseline eGFR and FI were added as covariates in all models except where they were the predictor. *p* < 0.05 considered significant, *p* < 0.20 warranted investigation of interactions

*Abbreviations*: *HR* hazard ratio, *CI* confidence interval, *FI* frailty index, *EQ-VAS* EuroQol 5D-5L visual analogue scale, *eGFR* estimated glomerular filtration rate in mL/min/1.73m^2^

^a^Data were split by time before commencement of dialysis versus time after commencement of dialysis

## Discussion

To our knowledge, this is the first study evaluating longitudinal frailty using a frailty index amongst older people with advanced, pre-dialysis CKD. We have demonstrated within this population that prevalence of frailty is high and increases substantially with time, with median FI transitioning from mildly frail to severely frail over four years [34]. The transition to worsening frailty was not influenced by commencing dialysis, and was also independent of age, sex and baseline eGFR and quality of life. Commencing dialysis at some point during the follow-up period was associated with better quality of life. Higher baseline FI and lower baseline eGFR predicted a higher risk of mortality, and commencing dialysis reduced the risk of mortality regardless of participants’ baseline FI.

The baseline prevalence of frailty (47%) observed in our study population is consistent with previous literature [1, 2], and is much higher than the reported prevalence of 17% in older Australian community-dwellers (where frailty was also measured by FI) [35]. The FI in our population at follow-up increased from a median of 0.21 at baseline to 0.43 after an average follow-up period of 4.2 years – an average increase in FI of 0.052 per year. In community-dwellers aged 65 and older, FI has been reported to increase by a mean of 0.02 per year [36]. There is a paucity of other literature regarding the trajectory of frailty in people with CKD, and no prospective studies have used a frailty index to assess frailty in this population.

In one study of 100 haemodialysis patients, frailty prevalence remained largely unchanged over one year follow-up at around 67% [37]. Another study of 762 haemodialysis patients observed some patients improved their frailty status while others declined over the 2-year follow-up period [38]. Only one study has evaluated frailty prospectively in the pre-dialysis setting (*n* = 56 participants) and found that after three years, the dialysis group had a lower proportion of frail patients compared to the conservative management group [39]. These studies all applied the Fried phenotype when assessing frailty. The Fried phenotype, though widely utilised to measure frailty in CKD settings [1], has limitations in its ability to quantify frailty beyond frail/non-frail dichotomisation. Additionally, the Fried phenotype is unidimensional in frailty assessment, focusing on a physical phenotype, and does not capture other deficits that may accumulate with time (such as cognitive impairment, alterations of sensorium and mood disturbances) which are important contributors to frailty [4].

Measurement of frailty as an index of accumulated deficits has many advantages. Unlike dichotomous frail/non-frail measurement tools, the FI, as a continuous measure, provides granular quantification of frailty. In situations where the prevalence of frailty is high, such as in CKD populations, the FI may enhance informed decision-making for individual participants by allowing precise quantification of their frailty. This issue is highlighted in our study – where the FI was dichotomised into frail/non-frail, the Kaplan–Meier survival curves were non-significant. However, when frailty was measured as a continuous variable it was found to be a significant predictor of mortality. The association between frailty and increased risk of mortality in patients with CKD is well documented [8], and may be mediated by multisystem dysregulation, inflammation and risk of infection [14].

Quality of life declined over time for participants assessed at follow-up, with a mean EQ-VAS of 70.0 at baseline and 63.0 at follow-up. The minimal clinically important difference in EQ-VAS is uncertain in the population included in this study, though has been reported to be 6.9 in patients with chronic obstructive pulmonary disease undergoing pulmonary rehabilitation [40]. Dialysis appeared to positively influence the trajectory of QOL, with those receiving dialysis maintaining their EQ-VAS scores while declining for participants not commencing dialysis. There is sparse literature examining the impact of dialysis versus conservative care pathways on QOL over time in older patients [41]. One previous study found no difference in QOL over time between dialysis and conservative care groups [42]. One study identified that quality of life remained stable in patients who underwent conservative management and declined in those who commenced dialysis [43]. One study identified, similar to ours, that quality of life remained stable over time in participants undertaking dialysis and declined in those undertaking conservative care [44]. Why dialysis positively influenced QOL in our study is uncertain and further studies to clarify this association would be valuable.

Our study demonstrated a survival benefit of dialysis with a 59% lower risk of mortality (hazard ratio 0.41) compared to conservative management during the study period. Whilst our study was not designed to address the survival benefit of dialysis in this population, our results are consistent with a recently published systematic review, observing universally across 18 longitudinal studies that conservatively managed participants had a higher mortality risk compared to those who received dialysis, with a pooled mortality hazard ratio of 0.47 in the dialysis group [45]. However, the survival benefit of dialysis was less clear in participants aged ≥ 80 and those with substantial comorbidities [45]. No prior studies have evaluated whether the survival benefit of dialysis is reduced if a participant is frail. Our study suggests that the survival benefit of dialysis is experienced regardless of the baseline FI of participants, though this requires further investigation in larger populations with adequate control of confounding factors.

### Limitations

Our study has several limitations, including small sample size, single-centre design and single reassessment of frailty. A reduced time to follow-up would have resulted in more participants still alive and thus more power when investigating changes in frailty and QOL over time. Our study did not differentiate whether a participant was not commenced on dialysis because they did not meet clinical indications to commence dialysis versus whether there was an active decision to pursue conservative management, and eGFR at the time of commencement of dialysis was not evaluated. Therefore, comparison of participants who received and did not receive dialysis is not truly reflective of equivalent time points and may introduce lead-time bias in survival analyses.

## Conclusion

Within the limitations of our study, our data suggest that frail participants received similar survival benefit from dialysis as non-frail participants, frailty of participants increased substantially whether dialysis was commenced or not, and commencing dialysis positively influenced the trajectory of QOL from baseline to follow-up. Larger studies are required to better understand the health outcomes of older patients with frailty undertaking dialysis versus conservative management.

### Supplementary Information

**Additional file 1:** **Supplementary Table 1.** Predictors of percent change in FI and QOL from baseline to follow-up assessment.

## Data Availability

The data underlying this article are available in the article and in its online supplementary material.

## Abbreviations

BMI: Body mass index
CHA: (InterRai) Community Health Assessment
CKD: Chronic kidney disease
eGFR: Estimated glomerular filtration rate
EQ-5D-5L: EuroQol 5-Dimension 5-Level Instrument
EQ-VAS: EuroQol Visual Analogue Scale
FI: Frailty index
HR: Hazard ratio
IQR: Interquartile range
QOL: Quality of life
RACF: Residential aged care facility
STROBE: Strengthening the Reporting of Observational Studies in Epidemiology
