# Supplementary material for: A prospective, observational study of frailty, quality of life and dialysis in older people with advanced chronic kidney disease
Source: BMC Geriatr. 2023 Oct 16;23:664. doi: 10.1186/s12877-023-04365-4 (PMC10580596; doi:10.1186/s12877-023-04365-4)
Supplement: Supplementary file 1 — Additional file 1: Supplementary Table 1. Predictors of percent change in FI and QOL from baseline to follow-up assessment. [file 12877_2023_4365_MOESM1_ESM.docx]

**SUPPLEMENTARY MATERIAL:**

A longitudinal study of frailty, quality of life and dialysis in older people with advanced CKD

**TABLE OF CONTENTS:**

Supplementary Table 1. Predictors of percent change in FI and QOL from baseline to follow-up assessment……………………………………………………………………………………………………………….…....2

**Supplementary Table 1. Predictors of percent change in FI and QOL from baseline to follow-up assessment**

|  | **Percent Change in FI** | | | **Percent Change in EQ-VAS** | | |
| --- | --- | --- | --- | --- | --- | --- |
|  | Coefficient | 95% CI | p-value | Coefficient | 95% CI | p-value |
| **Age (years)** | 0.37 | -1.22, 1.96 | 0.641 | 0.24 | -2.48, 2.96 | 0.858 |
| **Dialysis** | -12.28 | -33.01, 8.45 | 0.237 | 29.46 | 0.12, 58.80 | 0.049 |
| **Days on dialysis^#^** | 0.00 | -0.04, 0.03 | 0.789 | 0.02 | -0.03, 0.07 | 0.329 |
| **eGFR** | 0.49 | -2.93, 3.90 | 0.775 | -6.19 | -10.73, -1.65 | 0.009 |
| **Female** | -5.34 | -25.85, 15.16 | 0.600 | -10.01 | -40.00, 19.97 | 0.501 |
| **EQ-VAS** | -0.14 | -0.72, 0.44 | 0.618 |  |  |  |
| **Frailty index**  **(increasing 0.1 intervals)** |  |  |  | -8.87 | -25.92, 8.18 | 0.297 |

Abbreviations: FI, frailty index; EQ-VAS, EuroQol 5D-5L visual analogue scale; eGFR, estimated glomerular filtration rate in mL/min/1.73m^2^. #only for participants who commenced dialysis (n=35).
